# Our Prospects and Purposes

**Published:** 1887-10

**Authors:** 


### HALL'S JOURNAL OF HEALTH

TRUTH DEMANDS NO SACRIFICE ; ERROR CAN MAKE NONE.

VOL. 34. OCTOBER, 1887. NO. 10.

### OUR PROSPECTS AND PURPOSES.

It is now more than a year and a half since this Journal entered upon the liberal course, in relation to those moral and social questions which are more or less associated with every individual life. Its purpose has been in the past to deal with *facts* rather than *theories*, and to give its readers due information of the more prominent events and discoveries, which all persons should know and understand, without regard to their popularity or general acceptation. We are glad to be able to say at this writing, that our efforts have met with unexpected favor, even from those early patrons of the Journal, who have stood by it for so many years, whilst the enlargement of our subscription list and advertising patronage give assurances of an increased rather than diminished popularity. In the days of its greatest prosperity the average circulation was above 17,000, in the more troublous times it fell off to some extent, but is fast recovering its old standard. We shall not however be content to rest even there. The Journal ought to have 50,000 regular subscribers, and if our present patrons will exert themselves in its behalf, there is no manner of doubt but that it will reach those figures within a reasonable period. We do not ask this in view of any profit to the publishers, but in order that its pages of reading matter may be multiplied, and as a consequence its value as a monthly visitor greatly enhanced. This is not in a technical sense, a medical magazine, but rather a health journal, its chief object being to prevent so far as possible, the occurrence of those destructive maladies which beset mankind. To this end its efforts will be hereafter directed, hence, whatever adds to the comfort, cheerfulness and good order of the family circle will find a legitimate place in these columns

